# Assessment of thoracic spinal curvatures in static postures using spatially tracked 3D ultrasound volumes: a proof-of-concept study

**DOI:** 10.1007/s13246-022-01210-7

**Published:** 2023-01-10

**Authors:** Laura Meszaros-Beller, Maria Antico, Davide Fontanarosa, Peter Pivonka

**Affiliations:** 1 School of Mechanical, Medical and Process Engineering, Queensland University of Technology, Brisbane, Australia; 2 Centre for Biomedical Technologies, Queensland University of Technology, Brisbane, Australia; 3 Australian e-Health Research Centre, Commonwealth Scientific and Industrial Research Organisation (CSIRO), Queensland, Australia; 4 School of Clinical Sciences, Queensland University of Technology, Brisbane, Australia

**Keywords:** Ultrasound, Spinal landmarks, Spinal reconstruction, Spine phantom, Vertebral position

## Abstract

The assessment of spinal posture is a difficult endeavour given the lack of identifiable bony landmarks for placement of skin markers. Moreover, potentially significant soft tissue artefacts along the spine further affect the accuracy of marker-based approaches. The objective of this proof-of-concept study was to develop an experimental framework to assess spinal postures by using three-dimensional (3D) ultrasound (US) imaging. A phantom spine model immersed in water was scanned using 3D US in a neutral and two curved postures mimicking a forward flexion in the sagittal plane while the US probe was localised by three electromagnetic tracking sensors attached to the probe head. The obtained anatomical ‘coarse’ registrations were further refined using an automatic registration algorithm and validated by an experienced sonographer. Spinal landmarks were selected in the US images and validated against magnetic resonance imaging data of the same phantom through image registration. Their position was then related to the location of the tracking sensors identified in the acquired US volumes, enabling the localisation of landmarks in the global coordinate system of the tracking device. Results of this study show that localised 3D US enables US-based anatomical reconstructions comparable to clinical standards and the identification of spinal landmarks in different postures of the spine. The accuracy in sensor identification was 0.49 mm on average while the intra- and inter-observer reliability in sensor identification was strongly correlated with a maximum deviation of 0.8 mm. Mapping of landmarks had a small relative distance error of 0.21 mm (SD = ± 0.16) on average. This study implies that localised 3D US holds the potential for the assessment of full spinal posture by accurately and non-invasively localising vertebrae in space.

## Introduction

Analysis of spine biomechanics requires accurate measurement of vertebral position. In particular, inverse-kinematics analysis heavily relies on the assessment of vertebral position and spinal curvatures to derive the reciprocal angles of vertebral bodies. Currently, optoelectronic approaches are considered the gold standard for characterising gross spinal motion of the lumbar and/or thoracic spine [1]. Typically, markers are attached to the skin covering bony landmarks of the spine [2–5] and thorax [6, 7]. The position of individual vertebrae and their reciprocal angles is currently estimated as constant (population-based) fractions of the measured entire lumbar and/or thoracic segment angles using musculoskeletal models [8–10], recently reviewed in Alemi et al. [11]. However, this approach may not represent physiologic spine kinematics in all instances, neither does it allow for subject-specificity. Moreover, applied on the spine, concerns persist over the accuracy of skin-mounted techniques due to the lack of identifiable bony landmarks through palpation of the back that is limited to the spinous process. Typically, at least three landmarks of a rigid body are required to describe its position and orientation in space [12]. In addition, superficial soft tissue and highly individual skin movement artefacts relative to the underlying vertebra reduce the confidence in accurate landmark identification and tracking [13, 14].

On the other hand, imaging techniques allow for the visualisation of bony structures circumventing the aforementioned skin movement artefacts. Techniques based on X-ray [15, 16], bi-planar fluoroscopy [17–19] and upright magnetic resonance imaging (MRI) [20, 21] were able to provide reliable information on vertebral position. However, these imaging techniques are time-consuming, expensive and unlikely to gain access to on a daily basis as limited to a clinical environment. Moreover, X-ray and fluoroscopy expose the patient to ionising radiation increasing health risks which makes it unethical to be used in a ‘healthy’ cohort. Ultrasound (US) imaging is a sonographic imaging modality that allows for the visualisation and monitoring of organs in real-time [22]. Due to its benefits of being non-invasive, radiation-free and cost-effective, clinical applications using US imaging are rapidly advancing.

Applied on the back, US allows to visualise the posterior vertebral structures and has been largely driven by the clinical need to guide surgical interventions [23–25] and to quantify spinal deformities. For example, Chen et al. [26, 27] and Suzuki et al. [28] reported on the measurement of the spinous process and the laminae as landmarks for characterising the axial rotation in spinal deformities using US imaging. Recently, Brignol et al. [29] and Victorova et al. [30] proposed an automated method for vertebral landmark detection from US images. Results indicated that the detection of the laminae is more challenging compared to those of the spinous processes [29]. Two-dimensional (2D) tracked ultrasound (T-US) has previously been used for reconstructing the spine anatomy and measuring the curvature of scoliosis patients in the coronal plane [31–34]. Further, Cheung et al. [35, 36] and Zheng et al. [37] introduced a commercialised scoliosis assessment system *Scolioscan* for the three-dimensional (3D) spine reconstruction using freehand 2D T-US. *Scolioscan* was further used to study spinal curvatures in the coronal [38, 39] and sagittal plane [40] and was recently equipped with a robotic arm for the automatic learning-based localisation of the spinous process [30]. Those studies have shown that 2D US measures of curvature are comparable with those obtained using X-ray and MRI based imaging methods, however, de Reuver et al. [41] and Brink et al. [42] showed that reconstructions based on 2D US typically underestimate the Cobb angle, indicating potentially relevant errors in the US image registration procedure.

While these studies provide promising results, no attempts have been made for the spatial reconstruction of the spine and identification of spinal landmarks from volumetric 3D T-US imaging. Typically planar 2D T-US imaging is used in literature [31, 33, 38, 43] for the 3D reconstruction of the spine (i.e., a stack of 2D images) that (i) greatly depends on the accurate localisation of the 2D images (ii) requiring plane interpolation for a coherent 3D reconstruction. Moreover, since there is no exact feature overlap between adjacent 2D US images, it cannot be corrected through (manual or automatic) registration.

To overcome these limitations, this proof-of-concept study used for the first time a volumetric 3D T-US system for the assessment of static spinal postures on a benchmark level. It is hypothesised that volumetric 3D T-US enables a more facile 3D reconstruction of spinal anatomy and identification of landmarks in different spinal postures in a global coordinate system.

## Methods

**Fig. 1:**
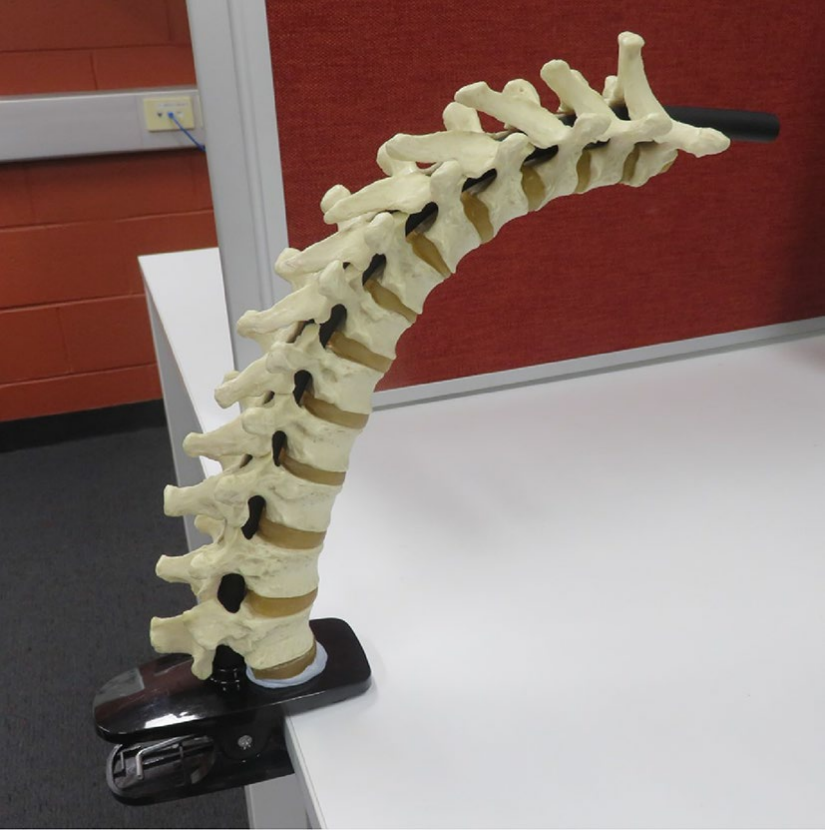
The phantom spine model (T1–T12) with a flexible rod inserted through the spinal canal

To evaluate the feasibility of using 3D T-US for the assessment of spinal postures, a phantom model of the thoracic spine depicted in Fig. 1 was used in the present proof-of-concept study. The phantom spine consisted of 12 individual vertebrae (T1–T12) connected by a string and intermediate rubbery intervertebral discs. The 3D US probe was tracked using commercially available electromagnetic sensors (NDI *Aurora*, see Sect. 2.1.2) in order to localise the volumetric US scans in global coordinates. The phantom spine was described in three static configurations (i.e., in different curvature poses) by registering multiple US volumes and three landmarks were identified on the posterior surface of each vertebra. The identification of landmarks was cross-validated through US-MRI registration using a MRI scan of the phantom.

### Data collection

#### MRI and US imaging of the phantom spine

The phantom spine model depicted in Fig. 1 (without rod) was immersed in water and scanned using MRI. A Siemens Magnetom Prisma 3T MRI system was used to produce T2-weighted images (TR: 3200 ms; TE: 410 ms) with an isotropic voxel resolution of $x=y=z=0.9~\mathrm {mm}$ of the phantom in a straight posture, i.e., with the vertebral levels aligned. In the obtained MRI scan, the bony surrogate of the spine was represented by dark pixel and the water by light pixel intensities, respectively.

Then, US scans were performed on the phantom spine using a linear 3D US transducer (Philips VL 13-5) with a volume field of view of $38~\mathrm {mm} \times 30^\circ$ and a voxel resolution of $0.18~\mathrm {mm} \times 0.18~\mathrm {mm} \times 0.34~\mathrm {mm}$. The settings of the Philips Epiq7 system optimised for the visualisation of the phantom model included: Frequency of 13 MHz; 4–6 cm penetration depth; emission power of − 0.5 dB; far field focus; dynamic range of 60 dB; medium persistence; wide scan; SonoCT real-time compound imaging technology and XRES image processing.

The spine phantom, reinforced by a flexible rod (see Fig. 1), was clamped onto a stable L-shaped base within the water tank and brought into three static configurations (i.e., poses) $pos_k$ with $k = 1, 2, 3$ in the sagittal plane by step-wise bending the model to three different configurations denoted as ‘straight’ ($pos_1$), ‘curved 1’ ($pos_2$) and ‘curved 2’ ($pos_3$) mimicking a spinal flexion. Due to the limited field of view in US, the spinal region of interest (T1–T5) could not be captured in a single US volume. Therefore, each configuration was captured by collecting 3D T-US scans of every vertebra between T1–T5 while the US probe stabilised by a probe holder was moved forward by a few centimetres ensuring a partial overlap between consecutive scans. Holding the probe head longitudinal to the phantom spine, further increased the overlap such that the resulting US scans included a whole vertebra and parts of adjacent vertebrae.

**Fig. 2:**
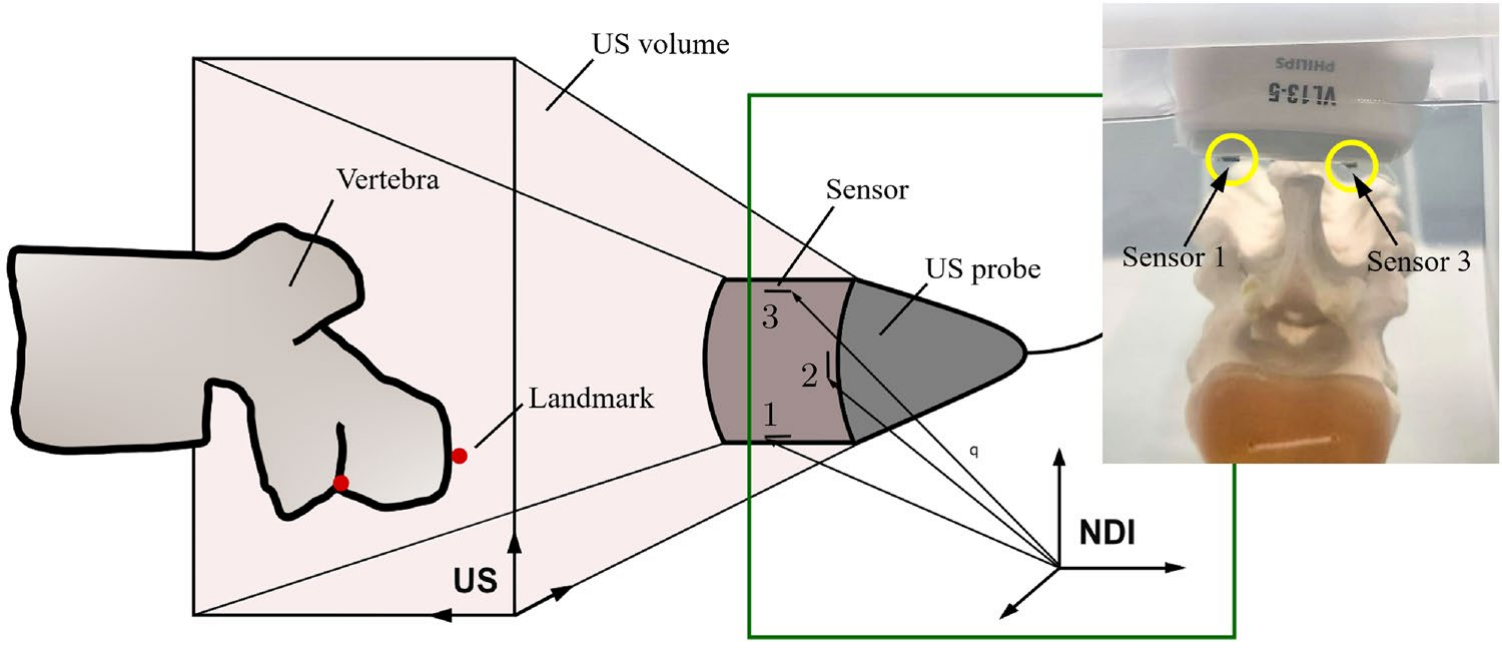
Illustration of the experimental data collection using T-US. The US probe was localised by three electromagnetic NDI tracking sensors attached to the scanning beam. Every voxel within the US volume (e.g., vertebral landmark) could be related to the location of the tracking sensors in the NDI coordinate system using coordinate transformation. Right: picture of the phantom spine during data collection with sensors (here: Sensor 1 and Sensor 3) indicated

#### Electromagnetic tracking of the US probe

During data collection, the US probe was localised using a commercial real-time electromagnetic tracking system, *Aurora* (NDI, Waterloo, ON, Canada). Three 6 degree of freedom rigid tracking sensors ($0.92~\mathrm {mm} \times 9.4~\mathrm {mm}$) were attached to the curved beam of the probe head using adhesive tape as shown in Fig. 2 and localised using the *Aurora* 20–20 Planar Field Generator ($0.7 ~\mathrm {mm}$ and $0.3^\circ$ tracking accuracy). At the time of US capture, the on-screen NDI data frame was saved such that every US scan could be related to the position and orientation of all sensors in the NDI logfile. As the sensors were positioned perpendicular to the $x{-}y$ and $y{-}z$ scanning plane of the US probe, they appeared as bright circular shapes in the respective plane when scrolling through the US volume along the *z*–axis and *x*–axis, respectively. The sensor tip coordinates were identified using *ImFusion* (ImFusion GmbH, Munich, Germany), a commercial software for the visualisation of medical images, in the last frame where the sensors were visible.

### Global localisation of the US volumes

Global localisation of the US volumes was done by relating the sensor tips identified in the US volumes to the sensor positions tracked by the NDI system. For every US volume, the global sensor tip coordinates $S_{i}^{\text {NDI}}$ of the three sensors were obtained through the NDI tracking system according to Equation (1).

1 $$\begin{aligned} S_{i}^{\text {NDI}} = (x_i,y_i,z_i) \end{aligned}$$

where the subscript $i = 1, 2, 3$ corresponds to the sensor ID and *x*, *y*, *z* corresponds to the sensor coordinates in the NDI coordinate system.

The sensor tip coordinates for the same sensors were also obtained in the US coordinate system by individually selecting the sensor tips for every US volume and configuration $\text {pos}_k$ in *ImFusion* as described in Sect. 2.1.2. These US-derived sensor positions $S_i^{\text {US}}$ were transformed with respect to the origin of the US coordinate system that was defined in the centre of the middle pixel of each US volume according to Equation (2).

2 $$\begin{aligned} S_{i}^{\mathrm{US},w} = (x'_i,y'_i,z'_i) \end{aligned}$$

where the subscript $i = 1, 2, 3$ corresponds to the sensor ID and $x'_i,y'_i,z'_i$ corresponds to the sensor tip coordinates in the ‘world’ US coordinate system. Further, the sensor positions were transformed to metric units (mm) using defined US voxel spacing.

In order to find the homogeneous transformation ${}_{NDI}{\mathbf {T}}^{US}$ from the US world coordinate system to the NDI coordinate system for each US volume, a least square regression method [44] in *MATLAB* R2021a (The MathWorks, Inc., Natick, MA, United States) was used. Using the obtained NDI ($S_{i}^{\text {NDI}}$) and US datapoints ($S_{i}^{\text {US},w}$) the regression method found the 3D rotation matrix ($R_{\text {3x3}}$) and the translation vector ($t_{\text {3x1}}$) of the homogeneous transformation by minimising the sum of squared distances between the two sets of datapoints according to Eqs. (3) and (4).

3 $$\begin{aligned} S_{i}^{\text {NDI}} = {}_{\text {NDI}}{\mathbf {T}}^{\text {US}}~S_{i}^{\text {US},w} \end{aligned}$$

with $${}_{\text {NDI}}{\mathbf {T}}^{\text {US}} = \left( \begin{array}{c|c} \begin{array}{ccc} &{} &{} \\ &{} R_{\text {3x3}} \\ &{} &{} \end{array} &{} t_{\text {3x1}} \\ \hline \begin{array}{ccc} 0 &{} 0 &{} 0 \end{array} &{} 1 \\ \end{array} \right)$$ and

4 $$\begin{aligned} \sum ^i \Vert R~S_{i}^{\text {US},w} + t - S_{i}^{\text {NDI}} \Vert ^2 \end{aligned}$$

where $S_{i}^{\text {US},w} = (S_{1}^{\text {US},w},S_{2}^{\text {US},w},S_{3}^{\text {US},w})$ and $S_{i}^{\text {NDI}} = (S_{1}^{\text {NDI}},S_{2}^{\text {NDI}},S_{3}^{\text {NDI}})$ are the sensor positions in the US (world) and NDI coordinates, respectively.

### Reconstruction of the spine

The anatomy of the thoracic spine T1–T5 was reconstructed by merging the acquired US volumes of each spine configuration (‘straight’, ‘curved 1’ and ‘curved 2’) in a two-step semi-automatic registration procedure starting with i) a NDI-based ‘coarse’ registration followed by ii) a ‘refined’ registration.

First, the US volumes capturing one configuration were visualised in a common coordinate system in *ImFusion*. Since homogeneous transformation in *ImFusion* maps the new coordinate system (i.e., NDI coordinate system) to the original coordinate system (i.e., US coordinate system), the inverse of the transformation matrix determined in Sect. 2.2  was applied to the respective volumes, resulting in a ‘coarse’ registration of the spine based on the NDI localisation.

Second, the ‘coarse’ registration was further refined using an automatic rigid registration in *ImFusion* by keeping the first US volume of the vertebral sequence static while the second volume was registered to the first one. The acquired change in translation and rotation was applied to all subsequent US volumes to keep their relative position constant. Next, the third volume was registered to the second volume, and the resulting relative transformation applied to the subsequent volumes until the vertebral sequence was complete.

### Identification and validation of landmarks in different configurations of the spine

**Fig. 3:**
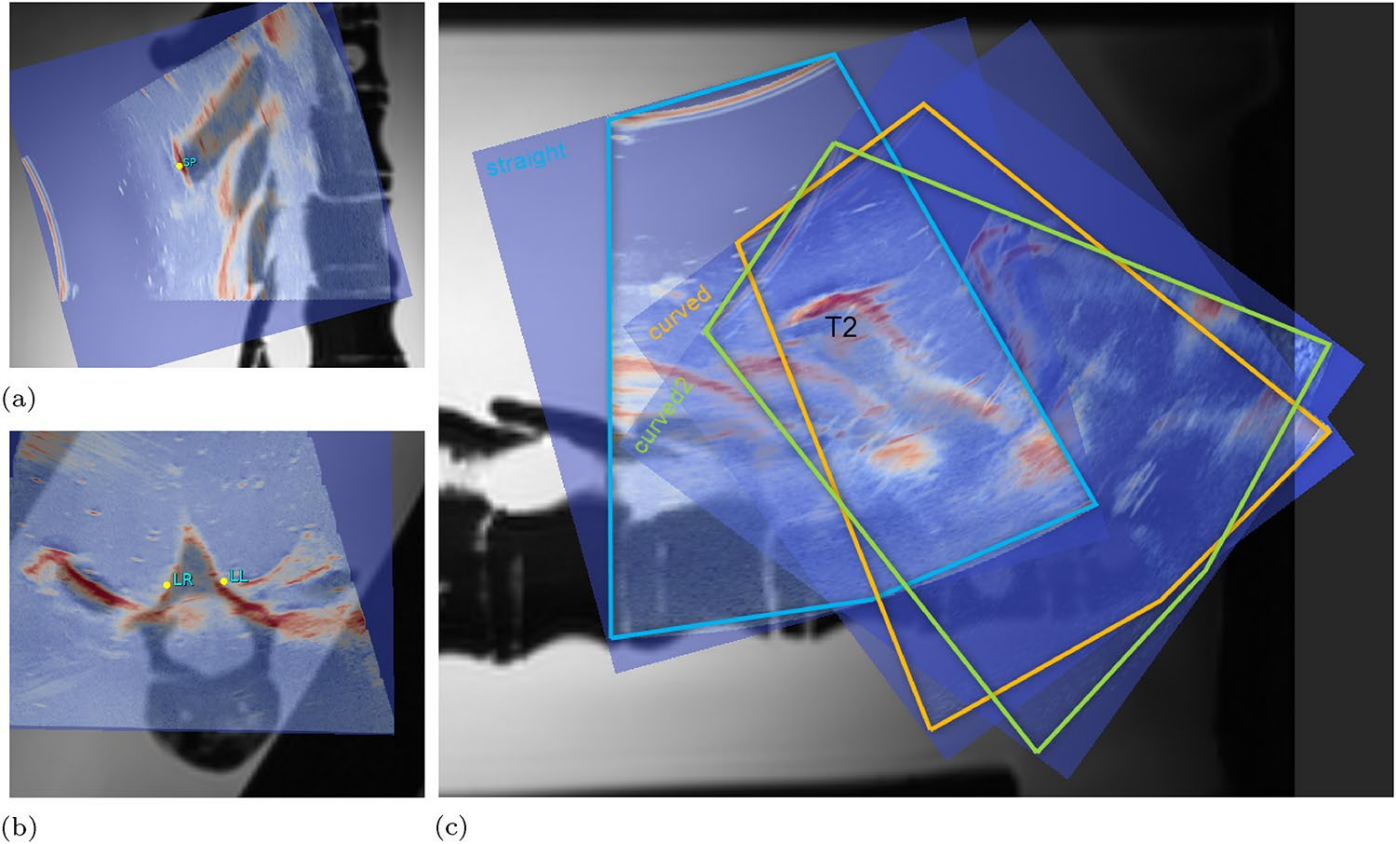
Landmark validation through manual US-MRI registration for the T2 vertebra in the sagittal plane in (**a**) and in the transverse plane in (**b**). Areas of strong US reflection are the spinous process (SPr) and the left (LL) and right lamina (LR), as indicated. In (**c**), all spine configurations $\text {pos}_k$ with $k = 1, 2, 3$ are visualised. The US-derived sensor tips $S_{\text {pos}_k}^{\text {US}}$ were selected individually for every US volume (straight: blue, curved 1: orange, curved 2: green) while US landmarks $L_{\text {pos}_k}^{US}$ were mapped from the straight configuration to the curved configurations

A minimum of three anatomical landmarks is required for the localisation of a body in a 3D space [12]. Previous studies have shown that US imaging allows for the visualisation of the posterior vertebral surface together with the identification of specific landmarks [29, 45]. Particularly, the tip of the spinous process (Fig. 3 (a)) and the laminae of the vertebral arch (Fig. 3(b)) create areas of hyper-echoic reflection in US.

A triplet of landmarks corresponding to the spinous process (SPr) and the left lamina (LL) and right lamina (LR) was selected in US coordinates for every vertebra between T1–T5 in the ‘straight’ configuration using *ImFusion*. Moreover, each US volume was co-registered to the MRI scan of the phantom spine in order to cross-validate the US-derived landmarks SPr, LL, LR. Note, each US-MRI registration performed in this study was done manually and verified by an experienced sonographer. In Fig. 3c the US-MRI registration for vertebra T2 is visualised for all configurations $\text {pos}_k$. Landmarks for the ‘curved 1’ ($L^{\text {US}}_{\text {pos}_2}$) and ‘curved 2’ configuration ($L^{\text {US}}_{\text {pos}_3}$) were derived from those selected in the ‘straight’ configuration ($L^{\text {US}}_{\text {pos}_1}$) by mapping the landmarks to the US volumes of the curved configurations. This ensured that the same landmarks were identified in the three configurations $\text {pos}_k$.

Similar to the transformation of sensor positions described in Sect.  2.2, the landmark coordinates were transformed relative to the centre of the middle pixel of each US volume ($L_{\text {pos}_k}^{\text {US},w}$) and further transformed to metric units (mm) using defined US voxel spacing. The homogeneous transformation ${}_{\text {NDI}}{\mathbf {T}}^{\text {US}}$ obtained in Sect.2.2  was reapplied to the US-derived landmarks to determine their location in the NDI coordinate system according to Equation (5).

5 $$\begin{aligned} L_{pos_k}^{NDI} = {}_\text {NDI}{\mathbf {T}}^{US}~L_{pos_k}^{US,w} \end{aligned}$$

### Error measurements

The evaluated errors included (i) the sensor identification error, (ii) the intra-observer and inter-observer reliability in sensor identification, (iii) the quantitative assessment of the obtained reconstructions, and (iv) the error introduced through landmark mapping. Note, all US-derived voxel coordinates were transformed to metric units (mm) before error calculation.

(i) The ‘coarse’ registration is dependent upon the accuracy in sensor identification from the US volumes (Sect. 2.4). Thus, the difference *E* of absolute distances *D* between sensor pairs $S_i S_j$ with $i, j = 1, 2, 3$ was computed according to Equation (6).
  6 $$\begin{aligned} E_{S_i S_j} = D_{S_i S_j}^{NDI}-D_{S_i S_j}^{US} \end{aligned}$$
  where $D_{S_i S_j}^{NDI}$ and $D_{S_i S_j}^{US}$ are the absolute sensor distances obtained from the NDI system and the US volumes, respectively.
(ii) The intra-observer and inter-observer reliability in sensor identification was analysed. US-derived sensor tip coordinates were selected for every US volume by two instructed observers. The intra-observer reliability in sensor selection was analysed for a single observer and compared to the selection of a second observer to obtain the inter-observer measures by computing the average of the absolute distance in sensor positions between the two respective selections.
(iii) The obtained ‘refined’ registrations for the three configurations were validated by an experienced sonographer with the possibility for modification, if required, to obtain a clinically acceptable reconstruction. To assess the reconstructions quantitatively, the relative spatial offset and the feature overlap between adjacent US volumes before and after refinement, was computed. The spatial offset was defined as the norm of the difference in translation (t) and rotation (R) between the NDI-based ‘coarse’ registration and the ‘refined’ registration according to Antico et al. [46]:
  7 $$\begin{aligned} t&= \sqrt{\Delta x^2 + \Delta y^2 + \Delta z^2} \end{aligned}$$
  8 $$\begin{aligned} R&= \sqrt{\Delta \alpha ^2 + \Delta \beta ^2 + \Delta \gamma ^2} \end{aligned}$$
  With $\Delta x = x_{REF} - x_{CR};~ \Delta y = y_{REF} - y_{CR};~ \Delta z = z_{REF} - z_{CR}$; $\Delta \alpha = \alpha _{REF} - \alpha _{CR};~ \Delta \beta = \beta _{REF} - \beta _{CR};~ \Delta \gamma = \gamma _{REF} - \gamma _{CR};$ where *x*, *y*, *z* and $\alpha , \beta , \gamma$ are the three translation and rotation values, respectively, obtained by the NDI-based ‘coarse’ registration (CR) and the ‘refined’ registration (REF) as indicated by the subscript. The feature overlap at the intersection of adjacent US volumes was assessed by segmenting the bony anatomy using an automatic threshold algorithm in *ImFusion* and comparing the dice similarity coefficient (DSC) and the volume overlap for both reconstructions.
(iv) To assess the error introduced through landmark mapping, the absolute distance between landmarks in the three different configurations $pos_k$ was compared. Landmarks obtained in the straight position were used as reference.

## Results

### Accuracy and reliability in sensor identification from US imaging

Table 1 shows the accuracy in sensor identification (i.e., selecting the sensor tip as described in Sect. 2.1.2) as the absolute difference in sensor distances between NDI-derived and US-derived sensor positions after coordinates were transformed from voxels to metric units (mm). Values ranged from 0.05 mm to 1.57 mm with an overall mean of 0.49 mm (SD = ± 0.41 mm). The highest uncertainty was between Sensor 1 and Sensor 2 ($S_1 S_2$) with an average deviation of 0.61 mm to the NDI data. Further results of this study include the intra- and inter-observer reliability in sensor identification representing the probability of selecting the same voxel as the sensor tip. The intra- and inter-observer reliability in sensor selection was comparable: average values ranged from 0.09 mm to 0.63 mm for a single observer and from 0.12 mm to 0.57 mm for two observers, respectively (Table 2). Overall, the sensor identification by the second observer showed less variance (SD range: ± 0.05 mm – ± 0.2 mm) than the selection of the first observer (SD range ± 0.05 mm– ± 0.6 mm). With respect to the NDI-derived sensor positions, both selections showed a similar deviation from tracked sensor distances of 0.49 mm (Table 1, first observer) and 0.58 mm (second observer).

**Table 1:** Absolute error in sensor distances ($S_1S_3$, $S_1S_2$, $S_2S_3$) between NDI-derived and US-derived sensor positions selected by the first observer in each configuration $pos_k$

$pos_k$	Absolute distance error		
	$S_1S_3$ (mm)	$S_1S_2$ (mm)	$S_2S_3$ (mm)
Straight			
T1	0.7	0.72	0.96
T2/3	0.3	0.24	0.18
T4/5	0.08	0.96	0.14
Mean (± SD)	0.36 (0.31)	0.64 (0.37)	0.43 (0.46)
Curved 1			
T1/2	0.13	0.08	0.4
T3/4	0.19	0.25	0.85
T5	0.2	0.88	1.33
Mean (± SD)	0.17 (0.04)	0.4 (0.42)	0.86 (0.47)
Curved 2			
T1–T3	0.44	0.29	0.11
T4	0.89	1.57	0.05
T5	0.3	0.56	0.49
Mean (± SD)	0.54 (0.31)	0.8 (0.67)	0.22 (0.24)
Overall mean (± SD)	0.49 (0.41)		

The vertebral levels captured in each US volume are indicated

**Table 2:** Intra- and inter-observer reliability in sensor selection

$pos_k$	Sensor 1			Sensor 2			Sensor 3		
	x	y	z	x	y	z	x	y	z
Intra-observer reliability (single observer)* (mm)									
Mean (± SD)	0.27 (0.16)	0.15 (0.14)	0.57 (0.43)	0.24 (0.05)	0.18 (0.24)	0.17 (0.3)	0.09 (0.09)	0.21 (0.14)	0.63 (0.6)
Straight	0.09	0.27	0.51	0.27	0.46	0	0.09	0.36	0.68
Curved 1	0.36	0	0.17	0.18	0	0	0.18	0.18	1.2
Curved 2	0.36	0.18	1.03	0.27	0.09	0.51	0	0.09	0
Inter-observer reliability (two observers)* (mm)									
Mean (± SD)	0.14 (0.09)	0.57 (0.07)	0.49 (0.13)	0.12 (0.06)	0.18 (0.06)	0.23 (0.2)	0.16 (0.04)	0.2 (0.09)	0.57 (0.3)
							(0.04)	(0.09)	(0.3)
Straight	0.12	0.61	0.34	0.06	0.18	0.34	0.18	0.3	0.23
Curved 1	0.06	0.61	0.57	0.12	0.12	0	0.12	0.18	0.8
Curved 2	0.24	0.49	0.57	0.18	0.24	0.34	0.18	0.12	0.68

These values show the confidence in sensor selection by a single observer (intra-observer reliability) and the probability of a second observer to select the same voxel (inter-observer reliability)

### Quantitative assessment of the spinal reconstructions

The experienced sonographer evaluated the ‘refined’ registrations to be accurate and comparable to clinical standards (Sect. 2.5). Given, anatomical features in adjacent US volumes were aligned, no modifications were needed, except for one registration in the ‘curved 2’ configuration, there was not sufficient anatomical overlap between two adjacent US volumes in order to perform the automatic registration. Hence, the volumes containing T1–T3 and T4 were excluded from the registration assessment.

**Fig. 4:**
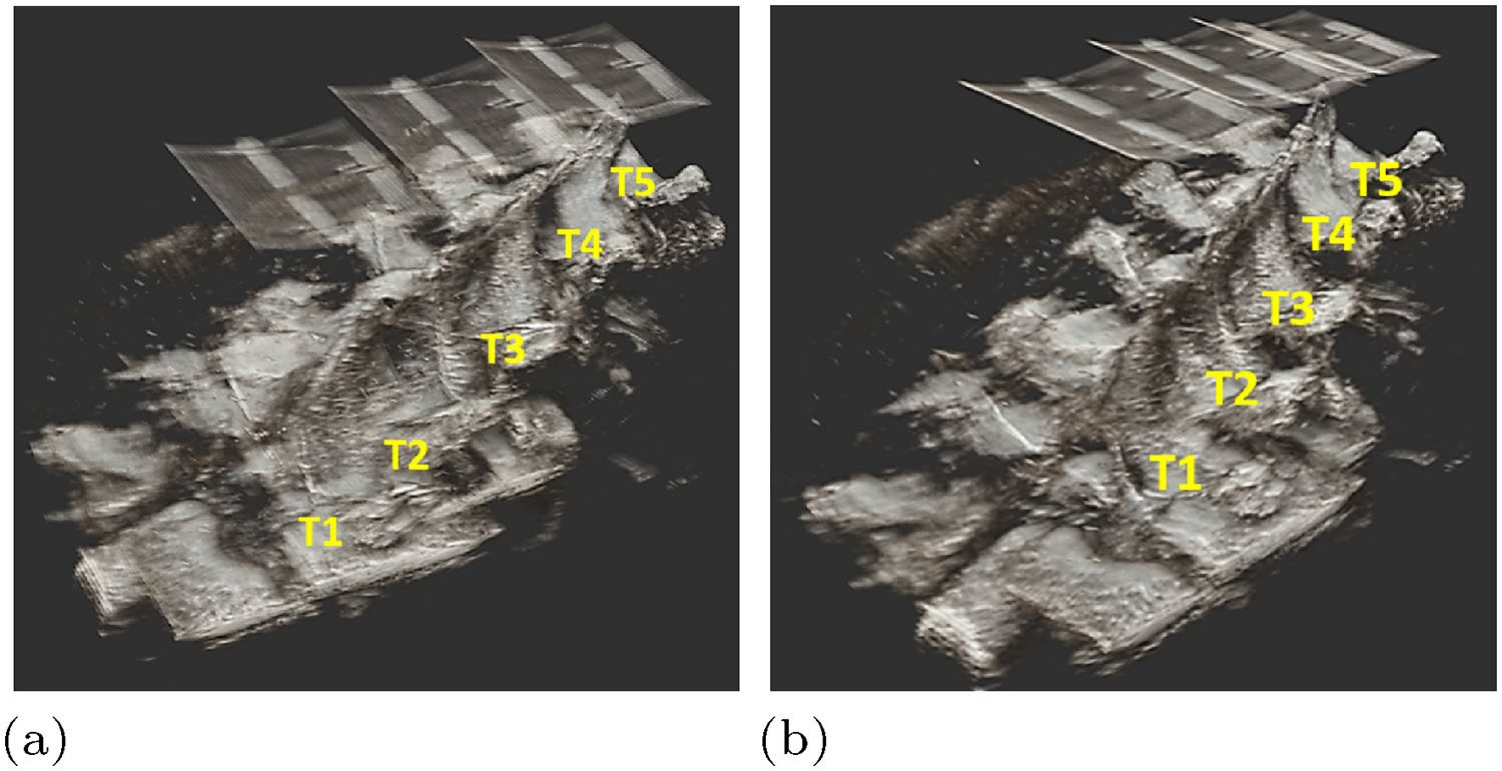
US-based anatomical reconstructions. 3D rendering of the thoracic spine (T1–T5) in the ‘straight’ configuration obtained in the NDI-based ‘coarse’ registration in (**a**) and the ‘refined’ registration in (**b**). The vertebral levels T1–T5 are indicated in yellow

**Fig. 5:**
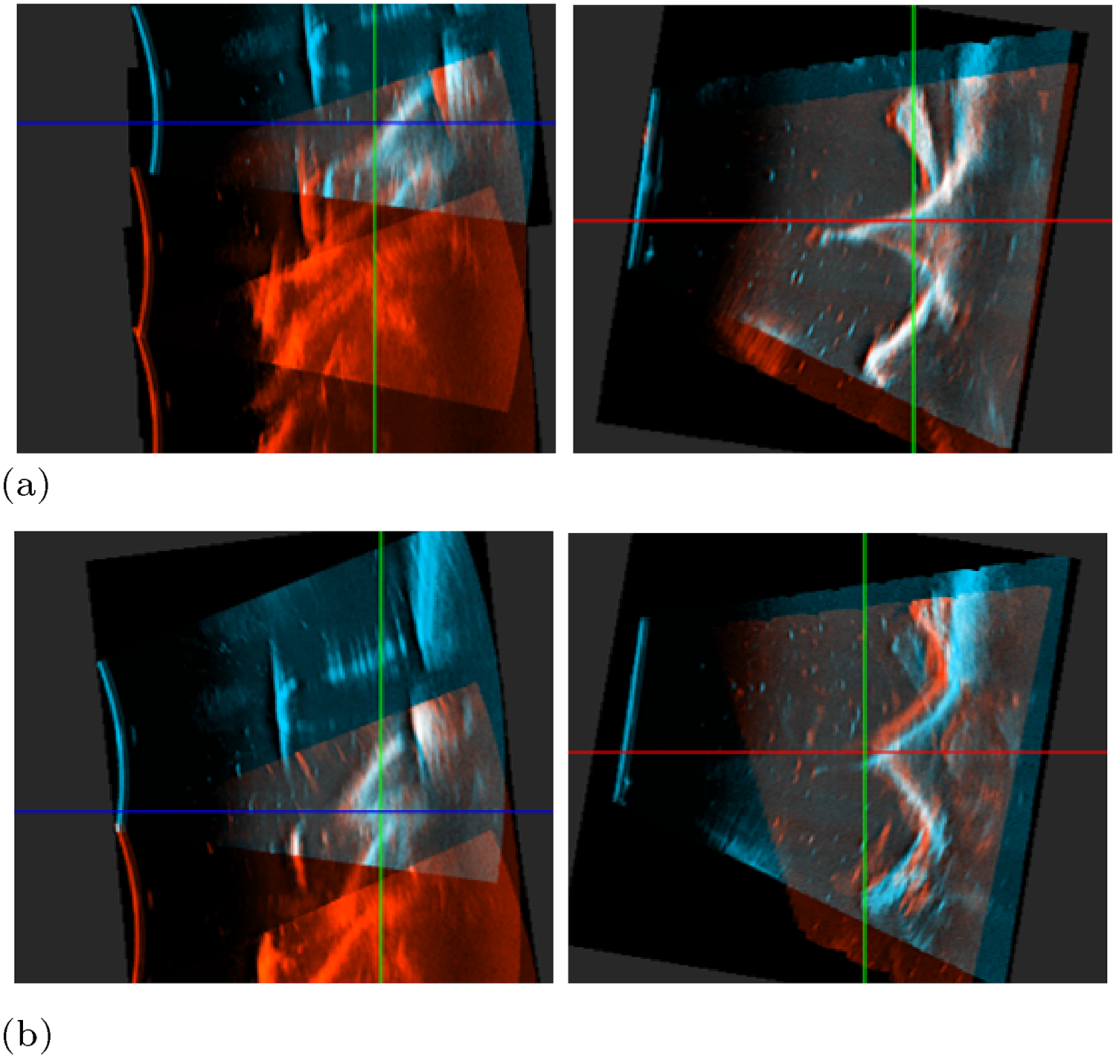
US overlap obtained with the NDI-based ‘coarse’ registration in (**a**) and with the ‘refined’ registration in (**b**). Each registration is shown in the sagittal (left) and in the transverse plane (right). The overlap of bony anatomy in the intersection of two adjacent US volumes (blue and red) is visualised in white

Figure 4 shows the spinal reconstructions for the ‘straight’ configuration generated by the NDI-based ‘coarse’ registration in (a) and the ‘refined’ registration in (b), respectively. Comparison of the two reconstructions shows an average normed difference in translation and rotation of 5.6 mm ± 2.4 SD (range 3.3–9.7 mm) and 3.6$^\circ$ ± 1.4 SD (range 1.3–5.2$^\circ$), respectively. Moreover, the overlap between adjacent US volumes increased in the ‘refined’ registration compared to the NDI-based ‘coarse’ registration. The improved overlap upon refinement is shown in Fig. 5 highlighting the intersection of adjacent US volumes. Table 3 shows the corresponding quantitative improvement of the ‘refined’ registration (REF) compared to the NDI-based ‘coarse’ registration (CR) through the DSC and the volume overlap computed on the bony segmentations at the intersection of adjacent US volumes. The relative DSC increased by up to +75% and the volume overlap increased by up to +72% with refinement.

**Table 3:** Quantitative comparison of the NDI-based ‘coarse’ registration and the ‘refined’ registration

$pos_k$	AbsDSC (%* )		RelDSC (%*)	Volume overlap (cm^3^)	
	CR	REF		CR	REF
Straight					
T1–T2/3	15.6	26.1	60	8.2	14.1
T2/3–T4/5	15.7	21	75	6.3	9.3
Curved 1					
T1/2–T3/4	4.5	11.5	39	1.9	4.7
T3/4–T5	1.4	3.5	40	0.5	1.3
Curved 2					
T1–T3/T4	–	–	–	–	–
T4–T5	6.1	16.7	37	0.8	2.2

$^{*}$Absolute (AbsDSC) and relative dice similarity coefficient (RelDSC), computed as AbsDSC(CR) / AbsDSC(REF). CR: ‘coarse’ registration; REF: ‘refined’ registration. The volume containing T1–T3/T4 in the ‘curved 2’ configuration was excluded from this assessment due to insufficient overlap

### Mapping landmarks in multiple configurations of the spine

**Fig. 6:**
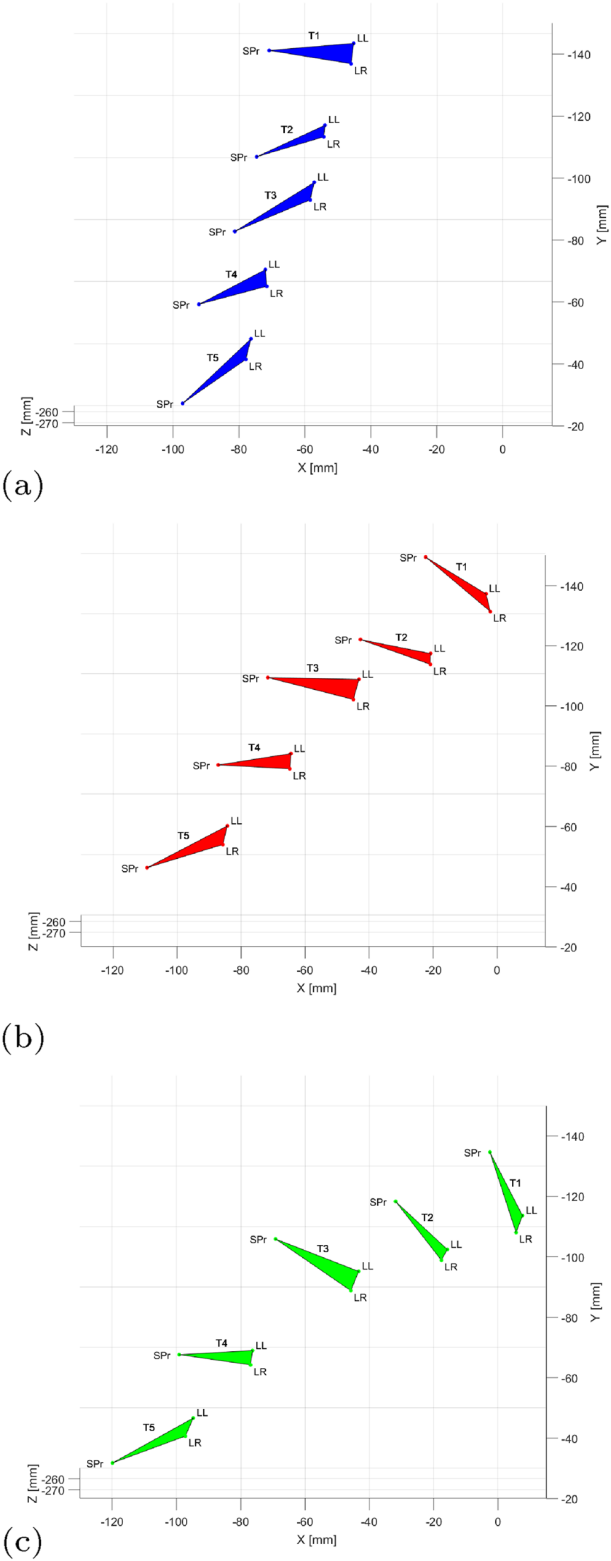
Mapping US-derived landmarks in three configurations of the spine $pos_k$ with $k=1,2,3$ mimicking a forward flexion. The level-specific triplet of landmarks SPr, LL, LR $\in L_{pos_k}^{NDI}$ describes a vertebra’s position in global NDI coordinates. Landmarks were selected for T1–T5 (indicated) in the ‘straight’ configuration in (**a**) and mapped to the curved configurations in (**b**) and (**c**). *SPr* spinous process, *LL* left lamina, *LR* right lamina

Figure 6 shows the three configurations $pos_k$ for levels T1–T5 based on US-derived spinal landmarks after transformation into global NDI coordinates as described in Sect. 2.4. Each vertebra’s position and orientation is described by the triplet of level-specific landmarks SPr, LL, LR $\in ~L_{pos_k}^{NDI}$ that were used to reconstruct the two-step flexion of the phantom spine from an initially straight to a curved configuration (Fig. 6). On average, the absolute distance between landmarks in the different configurations varied by 0.21 mm (SD = ± 0.16; range 0.01 mm–0.56 mm).

## Discussion

US imaging is among the most rapidly advancing medical imaging modality in the healthcare sector. Currently, US is the only radiation-free imaging modality allowing for the monitoring of organs in real-time [22]. However, applied on the spine, US was mainly used for image-guided spine interventions [24, 25] and for the assessment of spinal curvatures [31, 32, 34, 37, 38].

Currently, the kinematic assessment of the human spine is still an unsolved biomechanical challenge. To determine the location and orientation of vertebrae from externally is difficult due to the lack of identifiable bony landmarks. Moreover, applied on the spine, the potentially significant individual skin movement during motion tasks must also be considered. Although Mörl and Blickhan [21] have shown a high correlation between skin-mounted markers and the spinous process in the lumbar spine, large errors of up to 27 mm were observed elsewhere [13, 14].

Optoelectronic approaches, such as marker-based motion capture techniques are currently considered the gold standard to obtain gross spinal motion [2, 4], however, due to the aforementioned limitations, these methods cannot provide reliable intersegmental data for the spine. In fact, marker-based techniques are known to have system errors of up to 10 mm when studying human movement in general [47]. Considering the relatively short distance between the spinous process and the vertebral body, small errors in marker location can lead to significant errors in defining the position of the vertebra [14]. These errors will likely propagate and amplify over larger segments of the multi-joint structure of the spine such as the lumbar or thoracic region composed of multiple functional spinal units.

Towards advancing spinal motion assessment, this study used for the first time a 3D T-US system for the assessment of static spinal postures. This included the 3D reconstruction of the spinal anatomy of a phantom spine in three static configurations (i.e., curvature poses) and the identification of landmarks in these. In musculoskeletal modelling, for example, the reliable identification of three landmarks per segment [12] is of great importance as these are the kinematic input for inverse-kinematics and inverse-dynamics analysis. The major advantage of using T-US for the assessment of spinal postures is the possibility to localise a body in space while at the same time overcoming limitations such as soft tissue artefacts by directly visualising the bone.

During the experimental setup three small electromagnetic NDI tracking sensors were attached to the US probe and later identified in the US volumes in order to obtain information on the global position and orientation of the US volumes. Since the sensors were directly visualised and identified in the acquired US volumes, no calibration was needed for the tracking method used in this study. However, the localisation of the probe including sensor identification was the part of the developed approach most prone to error. As sensor position and the error resulting from it could not be assessed individually, the relative distance between US-derived sensor positions was computed and compared to NDI sensor data. The results in Table 1 show a high accuracy in sensor identification as the error measured between US-derived sensor tips and tracked sensor positions was small (0.49 mm on average). The largest deviation in sensor selection was 1.2 mm (Table 2) for a single observer while the inter-observer data show a strong correlation with a maximum deviation of 0.8 mm, proving that the proposed methodology is transferable between observers. An important factor contributing to the intra- and inter-observer errors is that, as a consequence of the high resolution of US images (voxel dimension: $0.18~\mathrm {mm} \times 0.18~\mathrm {mm} \times 0.34~\mathrm {mm}$), the sensor tips (diameter: $0.92~\mathrm {mm}$) are visible in multiple adjacent voxels. The sensor identification error could be avoided if the US system featured an integrated localisation of the probe [31, 33, 37].

Regarding the reconstruction of spinal anatomy, the 3D T-US imaging used in this study has two main advantages compared to other 2D T-US methods presented in the literature [31, 33, 38, 43]. First, the registration based on 3D US volumes is inherently more accurate than a stack of 2D T-US images of the same object. The 3D US probe scans the object in three different planes and directly generates a high-resolution US volume with visually relatable spatial features of the scanned object (i.e., vertebra). Second, with sufficient feature overlap between subsequent US volumes, a reconstruction purely based on US-US registration would be possible. In theory, the proposed 3D US approach, thus, can be independently used for anatomical reconstruction with or without tracking of the US probe. In this study, the initial ‘coarse’ registration provided through the NDI tracking system, enabled an automatic refinement in *ImFusion* based on intrinsic feature overlap of adjacent US volumes and the detection of anatomical landmarks in a common global coordinate system. Note, based on the validation by an experienced sonographer, the proposed approach of the NDI-derived ‘coarse’ registration followed by the automatic ‘refined’ registration together created US-based spinal reconstructions comparable to clinical standards. Thereby, no further validation of the ‘refined’ registration was necessary. As mentioned in Sect. 3.2, there was not enough overlap between the US volumes covering T1–T3/T4 in the ‘curved 2’ configuration to perform the automatic registration, hence, it was excluded from evaluation. To overcome this problem in the future, smaller increments along the spine during scanning would be necessary to obtain sufficient feature overlap in US scans covering adjacent vertebrae.

Challenges related to the presented approach include the automatic registration algorithm used in this study. Although robust to artefacts generated through water reflection, this method is likely reserved for phantom studies that provide a strong contrast and simplified anatomy (i.e., no ligament and tendon attachments). *In vivo*, surrounding soft tissue will influence the signal reflection intensity of US and cause additional artefacts, increasing the complexity of the registration problem. Other registration methods, most likely deep learning algorithms specifically trained for spinal application, will need to be introduced. However, this should not influence the visibility of bony landmarks selected in this study (i.e., spinous process and laminae) as these are reportedly well visible *in vivo* using US [23, 27, 31]. Another challenge related to the presented approach is the placement of the sensors attached to the scanning beam of the US transducer. To avoid manipulation through direct contact with the skin during *in vivo* scanning conditions, the sensors will need to be embedded, e.g., in a gel pad. Finally, for proof-of-concept purposes, anatomical reconstruction was performed for vertebrae T1–T5. This was considered sufficient to demonstrate the feasibility of the approach but can be extended to other vertebral levels of the spine.

Regarding landmark identification, automatic solutions could be considered in future, however, studies have shown limited success in terms of a robust extraction of the lamina [29, 48]. Thus, for more accurate results, mapping of landmarks as done in this study should be preferred over a landmark re-selection. This is supported by the small relative distance error of 0.21 mm (SD = ± 0.16) produced on average in this study (Sect. 3.3) that mapped landmarks initially selected in the ‘straight’ configuration to the other configurations (‘curved 1’, ‘curved 2’). Although considered to be of negligible magnitude, a possible explanation for this error may be that the landmark was mapped onto the border of two adjacent voxels in the US scan and was then shifted to the voxels’ centre coordinates. The time-consuming endeavour of US-MRI registration for landmark validation could potentially be avoided in future as no MRI-derived information was further used in this study. Alternatively, automatic US-MRI registration algorithms could be taken into consideration [49]. Generally, 3D US was, however, considered crucial for the correct identification of landmarks due to the partial overlap of vertebral structures (i.e., through the spinous processes) particularly in the thoracic region. In the 2D transverse image, prominent landmarks may belong to a different vertebra and could lead to a misinterpretation of bony features.

Ultimately, knowing the global position of three anatomical landmarks per vertebra, the relative location and orientation of adjacent vertebrae could be evaluated (i.e., under the assumption of rigid bodies) by reapplying the least-square optimisation method introduced in Sect. 2.2 on two sets of landmarks. The intervertebral angles were not evaluated given a phantom spine model was used in this study, that does not represent physiological bending properties.

While this study has shown promising results in the assessment of static spinal postures, more advanced scenarios could consider the assessment of dynamic spinal motion. On the human lower extremities, T-US has shown promising results in the kinematic assessment of the knee joint [50–52]. It was demonstrated that US showed lower kinematic errors compared to marker-based systems, with bone pins providing ground-truth data [50]. The potential of using dynamic US imaging for measuring lumbosacral motion in the sagittal plane was already demonstrated by van den Hoorn and colleagues [53] using 2D US. The change in lumbosacral angle was obtained on an *in vitro* human spine and on an *in vivo* porcine spine and was validated against video and fluoroscopy measurements. The same US system as used in this study could, for example, be used in a 4D mode to enable volumetric tracking of a specific vertebra. Dynamic analysis of the whole spine would be possible by attaching multiple US probes or even distributed flexible large scanning arrays to a participant’s back as recently done in Shea et al. [54]. In this respect, capacitive [55] or piezoelectric micromachined US transducers [56] can be used or even the recently introduced novel bioadhesive ultrasound device [22].

## Conclusion

Summarising, the present study has shown promising results in the spatial reconstruction of the anatomy and identification of vertebral landmarks on a phantom spine in different static postures using 3D T-US imaging. Given that US has the ability to directly and accurately image vertebral structures it has the potential to localise vertebrae in space non-invasively. However, due to the large number of articulations in the spine, capturing the whole spine is currently still very challenging and subject to further investigation.
